# Conjugate Addition of Indoles to *α,β*-Unsaturated Ketones Using a Brønsted Acid Ionic Liquid as an Efficient Catalyst

**DOI:** 10.3390/molecules14093222

**Published:** 2009-08-27

**Authors:** Chuan-Ji Yu, Chen-Jiang Liu

**Affiliations:** School of Chemistry and Chemical Engineering, Xinjiang University, Key Laboratory of Oil and Gas Fine Chemicals, Ministry of Education, 830046 Urumqi, China; E-mail: cjxyycj@126.com (C.-J.Y.)

**Keywords:** Brønsted acid ionic liquid, *β*-indolylketones, Michael addition

## Abstract

The Brønsted acid ionic liquid [PyN(CH_2_)_4_SO_3_H][*p*-CH_3_PhSO_3_] has been reported as an efficient catalyst for the Michael addition reaction of indoles to *α,β*-unsaturated ketones. Satisfactory results were obtained, with excellent yields and a simple experimental procedure. The catalyst could be recycled and reused up to three times without any noticeable decrease in the catalytic activity.

## Introduction

*β*-indolylketones have received much attention as important building blocks for the synthesis of various natural products and biologically active compounds [1]. Consequently, numerous methods have been developed for their synthesis using various catalysts such as Bi(OTf)_3_ [2], HfCl_4_ and ScCl_3_ [3], NO^+^BF_4_^-^ [4], CAN [5], InBr_3_ [6], PTSA [7], Bi(NO_3_)_3_ [8], GaCl_3_ [9], PVSA [10], Cu(OTf)_2_ [11], I_2_ [12], GaI_3_ [13], PDA [14], FAP [15], Zr(O^t^Bu)_4_ [16] and so on. However, most of the catalysts cannot be recovered and reused because they decompose under the quenching conditions. Furthermore, many of these reported protocols suffer from drawbacks such as strong acidic conditions, long reaction times and low yields of products. Recently, Gu *et al*. [17] reported silica-supported sodium sulfonate with ionic liquid as catalyst for Michael reactions of indoles. Although the yield was excellent, the reaction temperature is low and the catalyst can be recovered twice, the reaction time is too long. Therefore, to avoid these limitations, the introduction of a more efficient method affording higher yields is needed.

**Scheme 1:**
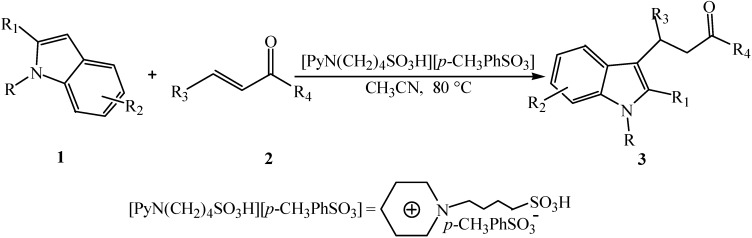
The synthesis of *β*-indolylketones catalyzed by [PyN(CH_2_)_4_ SO_3_H][*p*-CH_3_PhSO_3_].

In recent years, ionic liquids (ILs) have attracted increasing interest in the context of Green Chemistry owing to their advantageous properties such as negligible vapor pressure, high chemical stability, etc. ILs have gone far beyond the role of solvent, showing their significant importance as new catalysts in controlling the reactions in which they participate [18,19]. Recently, the synthesis of ‘‘task-specific’’ ILs with special functions according to the requirement of a particular reaction has become an attractive field [20,21]. To the best of our knowledge, there have been no reports on the use of Brønsted acid ionic liquids as catalysts for Michael additions of indoles. Herein we wish to report for the first time a novel, environment-friendly and efficient methodology for the synthesis of *β*-indolylketones by the reaction of indoles and *α,β*-unsaturated ketones using the SO_3_H-functionalized Brønsted acid ionic liquid [PyN(CH_2_)_4_SO_3_H][*p*-CH_3_PhSO_3_] as catalyst (Scheme 1).

## Results and Discussion

To evaluate the effect of the catalyst [PyN(CH_2_)_4_SO_3_H][*p*-CH_3_PhSO_3_] under different reaction conditions, the reaction of indole and chalcone was selected as a model reaction. The results are presented in Table 1. Initially the effect of solvent on the reaction was studied (Table 1, entries 1–4) and acetonitrile was found to be the best one. Next, we optimized the amount of [PyN(CH_2_)_4_SO_3_H][*p*-CH_3_PhSO_3_] required (Table 1, entries 4-8) and the optimum amount was found to be 10 mol%. In the absence of [PyN(CH_2_)_4_SO_3_H][*p*-CH_3_PhSO_3_], the reaction did not yield the desired product (Table 1, entry 5). The influence of the reaction time on the yield was also investigated (Table 1, entries 4, 9–14). It was clear that the highest yield was produced when the reaction time was 6 h, although the yield did not improve to any greater extent when the reaction time was increased from 4 h to 6 h. For the purpose of saving energy, we therefore chose 4 h as the reaction time. Hence, the best condition employs 0.1:1:1 mole ratio of [PyN(CH_2_)_4_SO_3_H][*p*-CH_3_PhSO_3_], indole and chalcone at 80 °C for 4 h using acetonitrile as solvent.

When optimizing the reaction conditions, the recycling of the catalyst in the reaction of indole and chalcone was investigated. After the separation of products, ethyl acetate was added to the filtrate, which split into two layers. The upper ethyl acetate layer contained unreacted raw material. The lower aqueous layer included the ionic liquid. The lower layer was concentrated on a rotary evaporator at 80 °C for 1 h under vacuum to remove excess solvent. [PyN(CH_2_)_4_SO_3_H][*p*-CH_3_PhSO_3_] could be recovered easily and directly reused in subsequent runs. As shown in Table 1, the desired product was obtained in 97, 93, 90% yields after 1–3 runs, respectively (entry 12^b^). This indicated that the ionic liquid [PyN(CH_2_)_4_SO_3_H][*p*-CH_3_PhSO_3_] was an efficient and recyclable catalyst for the conjugated addtion of indoles to *α, β*-unsaturated ketones.

To explore the generality and scope of this method, a wide variety of substituted indoles and *α,β*-unsaturated ketones were reacted with 10 mol% of [PyN(CH_2_)_4_SO_3_H][*p*-CH_3_PhSO_3_] catalyst under the optimized experimental conditions to afford the corresponding *β*-indolylketones in excellent yields. (Table 2). The results obtained indicated that the electron donating or withdrawing groups at the indole ring did not seem to affect the reaction significantly in terms of yields, nor do aromatic rings of *α,β*-unsaturated ketones.

**Scheme 2:**
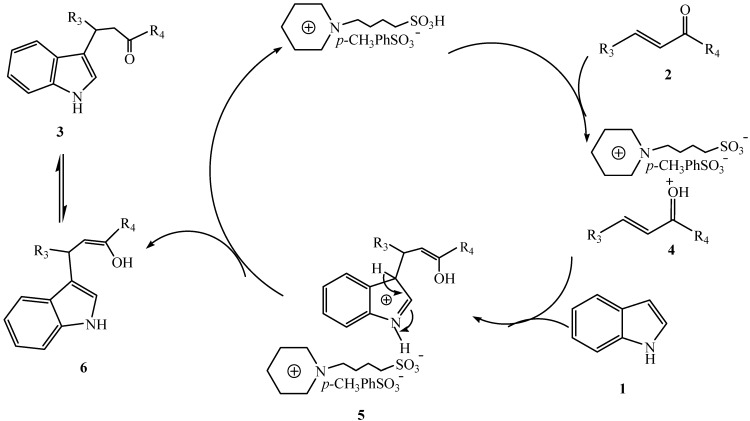
The proposed mechanism of synthesizing *β*-indolyketones catalyzed by [PyN(CH_2_)_4_SO_3_H][*p*-CH_3_PhSO_3_].

A plausible mechanism for the conjugate addition of indoles to chalcones is proposed in Scheme 2. [PyN(CH_2_)_4_SO_3_H][*p*-CH_3_PhSO_3_] coordinates with oxygen atom of *α,β*-unsaturated ketones (**2**) to give intermediate **4**. The electron rich *β*-position of the indole ring then attacks the electron deficient conjugated carbon-carbon double bond of **4** to afford **5**, followed by a hydrogen transfer to yield **6**. Finally **6** rearranged to give target compound **3** and [PyN(CH_2_)_4_SO_3_H][*p*-CH_3_PhSO_3_] catalyzes the next cycle.

## Conclusions

In conclusion, we have described for the first use of the Brønsted acid ionic liquid [PyN(CH_2_)_4_SO_3_H][*p*-CH_3_PhSO_3_] as an efficient catalyst for the synthesis of *β*-indolylketones. The procedure reported here has the advantages of mild reaction conditions, high yields of products, operational simplicity and catalyst recyclability.

## Experimental

### General

All compounds were characterized by IR, ^1^H-NMR spectra and elemental analysis. The IR spectra were obtained as potassium bromide pellets with a FTS-40 spectrometer (BIO-RAD, U.S.A). The ^1^H-NMR spectra were obtained on a Varian Inova-400 spectrometer using DMSO-d_6_ as solvent and TMS as an internal standard; chemical shifts are given in ppm. Elemental analyses (C, H, N) were performed on a Perkin-Elmer Analyzer 2400. Melting points were determined using a Büchi B-540 instrument. All melting points are uncorrected. The Brønsted acid ionic liquid [PyN(CH_2_)_4_SO_3_H][*p*-CH_3_PhSO_3_] was synthesized according to previous literature [22].

### General procedure for the synthesis of β-indolylketones

A mixture of indole or substituted indole (2 mmol), *α,β*-unsaturated ketone (2 mmol) and [PyN(CH_2_)_4_SO_3_H][p-CH_3_PhSO_3_] (0.2 mmol) was refluxed at 80 °C in acetonitrile (10 mL) for 4 h with stirring. The completion of the reaction was monitored by TLC. After cooling, the reaction mixture was poured onto crushed ice (30 g). The resulting precipitate was filtered under suction, and then recrystallized from ethanol to afford the pure product. The results are summarized in Tables 2. All products (except **3e, 3g, 3i, 3j, 3m**) are known compounds, which were characterized by mp, IR, ^1^H-NMR spectra, elemental analyses.

*3-(4-Chlorophenyl)-3-(1H-indol-3-yl)-1-(4-methoxylphenyl)propan-1-one* (**3e**): light yellow powder. Mp 202-205 °C; ^1^H-NMR δ: 3.74 (dd, 1H, *J* =7.6, 8.0 Hz, CH), 3.83 (s, 3H, OCH_3_), 3.86 (dd, 1H, *J* = 7.6, 8.0 Hz, CH), 4.85 (t, 1H, *J* = 7.6 Hz, CH), 6.88-8.00 (m, 13H, ArH), 10.83 (s, 1H, NH); IR (ν*_max_*; cm^–1^): 3383, 2971, 1657, 1599, 1489, 1256, 1180, 742; Anal. calcd. (%) for C_24_H_20_NO_2_Cl: C 73.94, H 5.17, N 3.59. Found: C 73.79, H 5.13, N 3.67.

*1-(4-Chlorophenyl)-3-(1H-indol-3-yl)-3-(4-methylphenyl)propan-1-one* (**3g**): light yellow powder. Mp 146-149 °C; ^1^H-NMR δ: 2.19 (m, 3H, CH_3_), 3.77 (dd, 1H, *J* = 7.6, 7.6 Hz, CH), 3.88 (dd, 1H, *J* =7.6, 7.6 Hz, CH), 4.80 (t, 1H, *J* = 7.6 Hz, CH), 6.85-8.03 (m, 13H, ArH), 10.83 (s, 1H, NH); IR (ν*_max._*; cm^–1^): 3418, 2914, 1674, 1587, 1455, 1244, 1179, 741; Anal. Calcd. (%) for C_24_H_20_NOCl: C 77.10, H 5.39, N 3.75. Found C 77.27, H 5.34, N 3.68.

*3-(4-Chlorophenyl)-1-(4-methoxylphenyl)-3-(2-methyl-1H-indol-3-yl)propan-1-one*(**3i**): brown powder. Mp 169-172 °C; ^1^H-NMR δ: 2.38 (s, 3H, CH_3_); 3.81 (s, 3H, OCH_3_); 3.87 (dd, 1H, *J* = 7.6, 7.6 Hz, CH), 3.90 (dd, 1H, *J* = 7.6, 7.6 Hz, CH), 4.88 (t, 1H, *J* = 7.6 Hz, CH), 6.82-8.19 (m, 12H, ArH), 10.74 (s, 1H, NH); IR (ν*_max._*; cm^–1^): 3312, 1664, 1596, 1464, 1258, 1169, 748; Anal. Calcd. (%) for C_25_H_22_NO_2_Cl: C 74.34, H 5.49, N 3.47. Found C 74.45, H 5.43, N 3.52.

*1-(4-Methoxylphenyl)-3-(2-methyl-1H-indol-3-yl)-3-(4-methylphenyl)propan-1-one* (**3j**): light yellow powder. Mp 172-175 °C; ^1^H-NMR δ: 2.2 (s, 3H, CH_3_); 2.36 (s, 3H, CH_3_); 3.80 (s, 3H, OCH_3_); 3.84 (dd, 1H, *J* =7.2, 7.6 Hz, CH ), 3.86 (dd, 1H, *J* = 7.2, 7.6 Hz, CH), 4.85 (t, 1H, *J* = 7.6 Hz, CH), 6.81-7.94 (m, 12H, ArH), 10.68 (s, 1H, NH); IR (ν*_max._*; cm^–1^): 3338, 1663, 1595, 1463, 1260, 1168, 747; Anal. Calcd. (%) for C_2__6_H_2__5_NO_2_: C 81.43, H 6.57, N 3.65. Found C 81.29, H 6.51, N 3.73.

*1,3-Diphenyl-3-(5-methoxyl-1H-indol-3-yl)propan-1-one* (**3m**): white powder. Mp 150-153 °C; ^1^H-NMR δ 3.67 (s, 3H, OCH_3_), 3.80 (dd, 1H, *J* =7.2, 7.6 Hz, CH), 3.90( dd, 1H, *J* =7.2, 7.6 Hz, CH), 4.82 (t, 1H, *J* = 7.2 Hz, CH), 6.66-8.02 ( m, 14H, ArH), 10.68 (s, 1H, NH); IR (ν*_max._*; cm^–1^): 3365, 1678, 1597, 1485, 1214, 1169, 723; Anal. Calcd. (%) for C_24_H_21_NO_2_: C 81.10, H 5.96, N 3.94. Found C. 80.93, H 6.01, N 3.90.

## Figures and Tables

**Table 1:** Effect of the catalyst [PyN(CH_2_)_4_SO_3_H][*p*-CH_3_PhSO_3_] under different conditions for the reaction of indole and chalcone.

Entry	Solvent	Catalyst (mol %)	Time (h)	Yield (%)^a^
1	EtOH	10	6	93
2	Ethyl acetate	10	6	88
3	THF	10	6	77
4	Acetonitrile	10	6	98
5	Acetonitrile	none	6	0
6	Acetonitrile	1	6	74
7	Acetonitrile	5	6	92
8	Acetonitrile	15	6	95
9	Acetonitrile	10	1	79
10	Acetonitrile	10	2	94
11	Acetonitrile	10	3	95
12^b^	Acetonitrile	10	4	97, 93, 90
13	Acetonitrile	10	5	97
14	Acetonitrile	10	7	95

*Reaction conditions*: indole (2 mmol), chalcone (2 mmol) and catalyst in solvent (10 mL), 80 °C; ^a^ Isolated yield; ^b^ Catalyst was recycled three times.

**Table 2:** [PyN(CH_2_)_4_SO_3_H][*p*-CH_3_PhSO_3_]-catalyzed synthesis of *β*-indolylketones.

Entry	R	R_1_	R_2_	R_3_	R_4_	Yields^a^ (%)	Mp (°C)^b^	
							Found	Reported (Lit.)
3a	H	H	H	Ph	Ph	97	126-129	127-128[17]
3b	H	H	H	Ph	4-Cl-Ph	96	153-156	151-152[9]
3c	H	H	H	Ph	4-CH_3_O-Ph	98	174-177	
3d	H	H	H	4-Cl-Ph	Ph	90	118-121	
3e	H	H	H	4-Cl-Ph	4-CH_3_O-Ph	90	202-205	
3f	H	H	H	4-CH_3_-Ph	Ph	98	132-134	130-131[9]
3g	H	H	H	4-CH_3_-Ph	4-Cl-Ph	94	146-149	
3h	H	H	H	4-CH_3_-Ph	4-CH_3_O-Ph	96	183-186	
3i	H	Me	H	4-Cl-Ph	4-CH_3_O-Ph	95	169-172	
3j	H	Me	H	4-CH_3_-Ph	4-CH_3_O-Ph	98	172-175	
3k	H	H	5-Br	Ph	4-Cl-Ph	98	189-191	188-189[13]
3m	H	H	5-CH_3_O	Ph	Ph	92	150-153	

*Reaction conditions*: indole or substituted indoles (2 mmol), *α, β*-unsaturated ketones (2 mmol), [PyN(CH_2_)_4_SO_3_H][*p*-CH_3_PhSO_3_] (0.02 mmol), acetonitrile (10 mL), 80 °C, 4 h; ^a^ Isolated yield; ^b^ Melting points were uncorrected.
